# Genetic variation in the flowering and yield formation of timothy (*Phleum pratense* L.) accessions after different photoperiod and vernalization treatments

**DOI:** 10.3389/fpls.2015.00465

**Published:** 2015-06-30

**Authors:** Venla Jokela, Ben Trevaskis, Mervi M. Seppänen

**Affiliations:** ^1^Department of Agricultural Sciences, University of Helsinki, HelsinkiFinland; ^2^Agriculture Flagship, Commonwealth Scientific and Industrial Research Organisation, Canberra, ACTAustralia

**Keywords:** flowering, photoperiod, *Phleum pratense* L., *PpMADS10*, *PpVRN1*, *PpVRN3*, timothy, vernalization

## Abstract

Timothy is a perennial forage grass grown commonly in Boreal regions. This study explored the effect of vernalization and photoperiod (PP) on flowering and growth characteristics and how this related to changes in expression of three flowering related genes in accessions from different geographic origin. Large variation was found in accessions in their vernalization and PP responses. In southern accessions vernalization response or requirement was not observed, the heading date remained unchanged, and plants flowered without vernalization. On the contrary, northern types had obligatory requirement for vernalization and long PP, but the tiller elongation did not require vernalization at 16-h PP. Longer vernalization or PP treatments reduced the genotypical differences in flowering. Moreover, the vernalization saturation progressed stepwise from main tiller to lateral tillers, and this process was more synchronized in southern accessions. The expression of *PpVRN1* was associated with vernalization while *PpVRN3* accumulated at long PP. A crucial role for *PpVRN3* in the transition to flowering was supported as in southern accession the transcript accumulated in non-vernalized plants after transfer to 16-h PP, and the apices transformed to generative stage. Differences in vernalization requirements were associated with variation in expression levels of *PpVRN1* and *PpVRN3*, with higher expression levels in southern type. Most divergent transcript accumulation of *PpMADS10* was found under different vernalization conditions. These differences between accessions can be translated into agronomic traits, such as the tiller composition of canopy, which affects the forage yield. The southern types, with minimal vernalization response, have fast re-growth ability and rapidly decreasing nutritive value, whereas northern types grow slowly and have better quality. This information can be utilized in breeding for new cultivars for longer growing seasons at high latitudes.

## Introduction

Timothy (*Phleum pratense* L.) belongs to *Poaceae* family, which includes some of the world’s most important cereal and pasture grasses (Stewart et al., 2011). It is a perennial cool-season forage grass that is commonly used in grass leys in temperate regions. It is well adapted for cultivation in Boreal climate due to its winter hardiness and adaptation to wide range of PP conditions (e.g., Andrews and Gudleifsson, 1983; Jönsson et al., 1992; Stewart et al., 2011). The level of genetic diversity in timothy accessions of different origin is high (Tanhuanpää and Manninen, 2012; Fjellheim et al., 2015). High levels of genetic variation are also found, within individual accessions, likely due to the wind pollinating outcrossing behavior and hexaploid nature of timothy (Tanhuanpää and Manninen, 2012).

There is considerable variation in flowering behavior amongst different accessions of timothy. Fiil et al. (2011) have showed that heading time (HD) is correlated to geographical origin of timothy. One component of the variation in flowering behavior is differences in vernalization requirement; the need for prolonged winter cold to stimulate flowering (Fiil et al., 2011; Jokela et al., 2014). When tested at natural Danish day length conditions (15–18 h), Fiil et al. (2011) identified considerable variation in vernalization response for timothy accessions of different origins, collected from latitudes between 35° N and 70°N. Vernalization accelerated flowering and development of timothy in all tested cultivars and accessions (Seppänen et al., 2010; Fiil et al., 2011; Jokela et al., 2014). For two Finnish timothy cultivars, ‘Iki’ (originated 63°N) and ‘Tuure’ (bred for cultivation 60–64°N), 10 weeks vernalization was required for proper flowering, although more southern ‘Tuure’ was able to flower also without vernalization in 16-h PP (Jokela et al., 2014). There exists wide genetic diversity in timothy cultivars and accessions for PP sensitivity (Heide, 1982; Junttila, 1985). For this study, PP treatments were selected based on the earlier results, where 12 h was shown to delay flowering (Jokela et al., 2014), and since the critical PP for accelerating flowering of timothy is typically being between 14 and 18 h (Heide, 1982). The length of vernalization was chosen based on previous studies of the effects of 0–15 weeks vernalization on flowering of timothy (Seppänen et al., 2010; Fiil et al., 2011; Jokela et al., 2014).

Timothy canopy is comprised of three different tiller types of different ages (Seppänen et al., 2010; Gustavsson, 2011; Virkajärvi et al., 2012; **Supplementary Figure S1**). The stem formation has an important role in the spring yield formation, because the vernalized spring growth consists mainly of stem producing GEN and ELONG tillers, which have a true stem with palpable nodes and apex at vegetative stage and high from soil surface (Virkajärvi et al., 2012). The second and third yields are composed of more vegetative tillers and leaves, and then leaf characters have larger importance (Virkajärvi et al., 2012). The stem producing GEN tillers are heaviest and thus, can contribute more to the silage yield than ELONG or VEG tillers (Virkajärvi et al., 2012). In forage grasses flowering was thought to be strictly associated with rapid decrease of stem digestibility. Our earlier study showed, however, that the lignified sclerenchyma ring forms similarly in time to all stem forming tillers irrespective of the developmental stage of apex (Seppänen et al., 2010). Thus, the ideotypic timothy canopy would consist of heavy stem forming tillers where the lignified sclerenchyma ring has not yet fully developed. In addition, an ideotypic timothy genotype would produce stem forming tillers rapidly without vernalization throughout the growing season. Doing so, the critical LAI could be high in all harvests and thus, the biomass accumulation would reach its maximum.

In winter cereals the saturation of vernalization requirement can be measured as FLN (Berry et al., 1980). Mahfoozi et al. (2001) noticed that in wheat plants where vernalization requirement was not fulfilled, the apex remained longer at vegetative stage producing new leaves instead of generative structures, which was seen as higher FLN. When vernalization requirement is saturated, the number of leaves will remain at constant level even when vernalization time is extended (Mahfoozi et al., 2001). In forage grasses the connection of FLN and vernalization saturation has not completely understood. Understanding the relationship between FLN and vernalization would bring important information on the development of the growth characteristics of different tiller types.

Variation in vernalization and PP requirements is important for breeding timothy cultivars with flowering behaviors suited to different geographical regions. How this variation can impact on other aspects of agronomic performance, e.g., tiller composition and yield formation under different PP or vernalization conditions has not yet been resolved. In timothy breeding programs, genetic variation between accessions of different origin has traditionally been utilized to combine desired traits (Tanhuanpää and Manninen, 2012). Southern accessions tend to grow faster after cutting (Larsen and Marum, 2006), whereas northern accessions have better winter hardiness (Jørgensen et al., 2010). Rapid stem growth is associated with development of lignified sclerenchyma ring and decrease in the digestibility of the stem (Seppänen et al., 2010). Therefore, the use of southern accessions in breeding programs accelerates growth, but can also decrease the nutritive value of the harvested forage yield.

The simplified model of molecular flowering-time regulation by vernalization and PP in monocots has been reviewed by several authors (e.g., Distelfeld et al., 2009; Kamran et al., 2014; Fjellheim et al., 2014). *VERNALIZATION1* (*VRN1*) is a major vernalization response gene in several studied monocots (e.g., Hemming et al., 2008; Sasani et al., 2009) and similar to *APETALA1*(*AP1*) in *Arabidopsis* (Kaufmann et al., 2010), whereas *VRN3* is an integrator of the vernalization and PP pathways, and a strong flowering promoter (Yan et al., 2006), and similar to *FLOWERING LOCUS T* (*FT*) in *Arabidopsis* (Kardailsky et al., 1999). In cereals flowering is repressed by *VRN2* (Hemming et al., 2008), and in grasses (e.g., *Lolium perenne)* by *LpMADS10* homologs (Ciannamea et al., 2006) under SD conditions. Seppänen et al. (2010) showed that vernalization genes, especially *VRN1*, are regulated similarly in timothy as in cereals, and the apex transition from vegetative to reproductive stage is connected to the expression peak of *VRN1* also in timothy. *PpVRN1, PpVRN3*, and *PpMADS10* genes were also identified and the expression patterns were shown to be similar to equivalent genes in other *Poaceae* species (Jokela et al., 2014).

Ecotypic variation in vernalization requirement of flowering has been reported in many species including *Arabidopsis thaliana* (Grillo et al., 2013), *A. lyrata* (Quilot-Turion et al., 2013), barley (Loscos et al., 2014), *Brachypodium distachyon* (Ream et al., 2014), *Poa* sp. (Heide, 2001), and wheat (Iwaki et al., 2001). Mutations, insertions or deletions in the promoter of first intron of *VRN1* may result rapid flowering without vernalization (Yan et al., 2004; Fu et al., 2005), and generally for spring growth habit. Also variations in *VRN3* due to copy number or structural rearrangements can have effect on the flowering time of cereals (Yan et al., 2006; Chen et al., 2013). At epigenetic level winter types are more methylated compared to spring types (Sherman and Talbert, 2002). The structural changes in *FRIGIDA* (*FRI*; Johanson et al., 2000) and *FLOWERING LOCUS C* (*FLC;* Grillo et al., 2013), provide an example of genetic variation underlying the ecotypical differences in *Arabidopsis*, whereas in cereals epigenetic changes in *VRN1* (Oliver et al., 2009) and in *FT/VRN3* (Diallo et al., 2012) explain differences. To date it has not been able to show any clear epigenetic changes in cereal’s flowering repressor *TaVRN2* (Diallo et al., 2012).

The objective of this study was to investigate how timothy accessions from different geographic origin respond to vernalization and PP treatments relative to growth characteristics, and how these differences can be translated into agronomic parameters. These differences were characterized at molecular level by studying the expression of major vernalization genes in relation to release of stem growth and flowering.

## Materials and Methods

### Plant Material and Experimental Conditions

Fiil et al. (2011) tested 38 timothy accessions for vernalization responses, and three of those accessions with strong (NGB14403, NGB 17198, 51998), one with intermediate (6116) and two with weak vernalization response (NGB4053, PI204909) were selected for this study (**Table 1**). In addition, two breeding lines of northern (BOR N) and southern (BOR S) origin and cultivars characterized previously for their agronomic performance in Boreal climate (Grindstad, Donatello, BOR1) were included.

**Table 1:** Eleven timothy (*Phleum pratense* L.) accessions used in this study.

Accession name	Origin	Accession classification	Vernalization response as defined by Fiil et al. (2011)	Material type, accession number*	Experiment used
BOR S	Czech	Southern	Unknown	Breeding line, BOR	1, 2
BOR N	Finland	Northern	Unknown	Breeding line, BOR	1, 2
Donatello	Finland	Southern, continental	Unknown	Commercial cultivar, BOR	1
BOR1	Finland	Southern, marine	Unknown	Breeding line, BOR	1, 2
Grindstad	Norway	Southern	Unknown	Commercial cultivar, Maatalouskesko	1, 2
Närekumpu	Finland	Northern	Strong	Landrace, NGB14403	1
Karasjok	Norway	Northern	Strong	Wild or semiwild, NGB17198	1
Saltum	Denmark	Southern	Weak	Wild or semiwild, NGB4053	1
Turkey	Turkey	Southern	Weak	Wild, GRIN, PI204909	1
6116	Italy	Southern	Intermediate	Uncertain, KEW6116	1
51998	England	Southern	Strong	Uncertain, KEW51998	1

*BOR- Boreal Plant Breeding, Finland; Maatalouskesko, Finland; NGB-Nordic gene bank, Alnarp, Sweden; GRIN- Germplasm Resources information network, Beltsville, MD, USA; KEW- Royal Botanic Gardens, Kew, UK.

Timothy accessions were tested for vernalization and PP responses in two separate experiments (**Table 1**). The propagation of the plant material was timed so that vernalization treatments finished at the same time, and plants with different treatments were transferred to greenhouse together. In the first experiment plants were propagated on 14.10.2010, 4.11.2010, 18.11.2010, 6.1.2011, and 20.1.2011, and transferred to greenhouse on 17.2.2011. In the second experiment plants were propagated on 17.11.2011, 21.12.2011, and 21.2.2012, and transferred to greenhouse on 21.3.2012. Clonal material was propagated using vegetative plants (stages 22–28; Gustavsson, 2011) and grown for 4 weeks in SD conditions (20/15°C day/night, light intensity was between 200 and 500 μmol m^-2^s^-1^, 12 h day length) in fertilized and limed peat (Kekkilä B2, Finland).

Clonally propagated plants having one to two leaves (and a single vegetative apex) were transferred to growth chamber (Weiss Technik, Germany) and vernalized at 6/4°C (day/night) and 8-h PP for 0, 2, 10, 12, or 15 weeks (Experiment 1) and 0, 10 or 15 weeks (Experiment 2). In both experiments, non-vernalized plants were grown in SD conditions (20/15°C day/night, light intensity was between 200 and 500 μmol m^-2^s^-1^, 12-h PP) for 4 weeks, and transferred to the LD greenhouse simultaneously with all vernalized plants. Thus, there was difference in the PP conditions for the control and vernalized plants, but based on earlier studies we assume that this should not have any effect on the results, because the PP after vernalization should have more important effect. In growth chamber, pots with three individual plants were arranged in a completely randomized design with three replicates, and rotated once a week in order to minimize the effects of possible environmental variation within the growth chamber. After vernalization plants were transferred at the same time with non-vernalized plants to greenhouse (20°C/15°C day/night, light intensity was between 200 and 500 μmol m^-2^s^-1^), to 16 h PP (Experiment 1), and to 12, 16, or 20 h PP (Experiment 2) conditions.

Flowering was expressed as HD which was recorded for individual plants separately when the first spikelets had emerged. Plants which did not produce flowering tillers at all, a set value of 200 days was given for the calculations. The height of the three individual main tillers in three (Experiment 1) or two (Experiment 2) replications were measured after 3 weeks’ time in greenhouse conditions. FLN, the type of main tiller, and the number and type of lateral tillers were calculated at the end of the experiments, ca. 100 days in greenhouse conditions. Tiller types (VEG, ELONG, GEN) were defined as described in Jokela et al. (2014; **Supplementary Figure S1**).

Leaf material for RNA samples were collected 0, 7, and 28 days after the transfer to greenhouse (Experiment 1), or 0 and 14 days after the transfer (Experiment 2). The developmental stages of the apical meristem of three individual plants were determined at RNA sampling time. The scale of apical meristem by Sweet et al. (1991) classifies apices as vegetative (stages 1–2), with double ridge stages (stages 3–4), and as generative (stages 5–11). The sampling and determination of apices was conducted as removing the plant tissue (leaves and part of the stem) around the apex from three individual plants. The developmental stage was observed using light microscope.

### Gene Expression Analysis

Total RNA was extracted from timothy leaves from both experiments using Pine Tree Method (Chang et al., 1993) and diluted in 12 μl of DEPC- water. RNA was cleaned up using NucleoSpin XS (Macherey-Nagel, Düren, Germany) kit according to manufacturer’s instructions and diluted in 10 μl of DEPC- water. cDNA synthesis with DNase treatment was performed using the SuperScript III (Invitrogen, Karlsruhe, Germany) one-step cDNA kit, where 1 μg of total RNA was used per reaction, according to the manufacturer’s instructions.

Quantitative real time PCR analysis was conducted in a Roche Light Cycler 480 (Roche Applied Science, Basel, Switzerland) in a 96-well plate one reaction containing 0.5 μM forward and reverse primer (actin, *PpVRN1*, *PpVRN3*, *PpMAS10*; **Supplementary Table S1**), 10 μl SYBR Green master mix (Roche, Basel, Switzerland), 3 μl water and 5 μl diluted cDNA. The PCR program was: an initial denaturation step at 95°C for 10 min, following 40 cycles of 10 s at 95°C, 20 s at 59°C and 30 s at 72°C. Expression levels were calculated by the 2^-ΔΔCt^ method (Livak and Schmittgen, 2001) actin for normalization of the data, and results were reported as a fold change relative to a calibrator sample (non-vernalized BOR S and BOR N samples). The efficiency for the primers was calculated using a decreasing series of cDNA concentrations, and all primer efficiencies were close to 2.

### Statistical Analyses

Analysis of variance (ANOVA) was performed using PASW 18 software (SPSS Inc., Chicago, IL, USA; replicates as random effects, and environmental and accessions, and their interaction were as fixed effects), and multiple comparisons of means were performed with Tukey’s test.

## Results

### Genetic Variation of Vernalization Response

**FIGURE 1:**
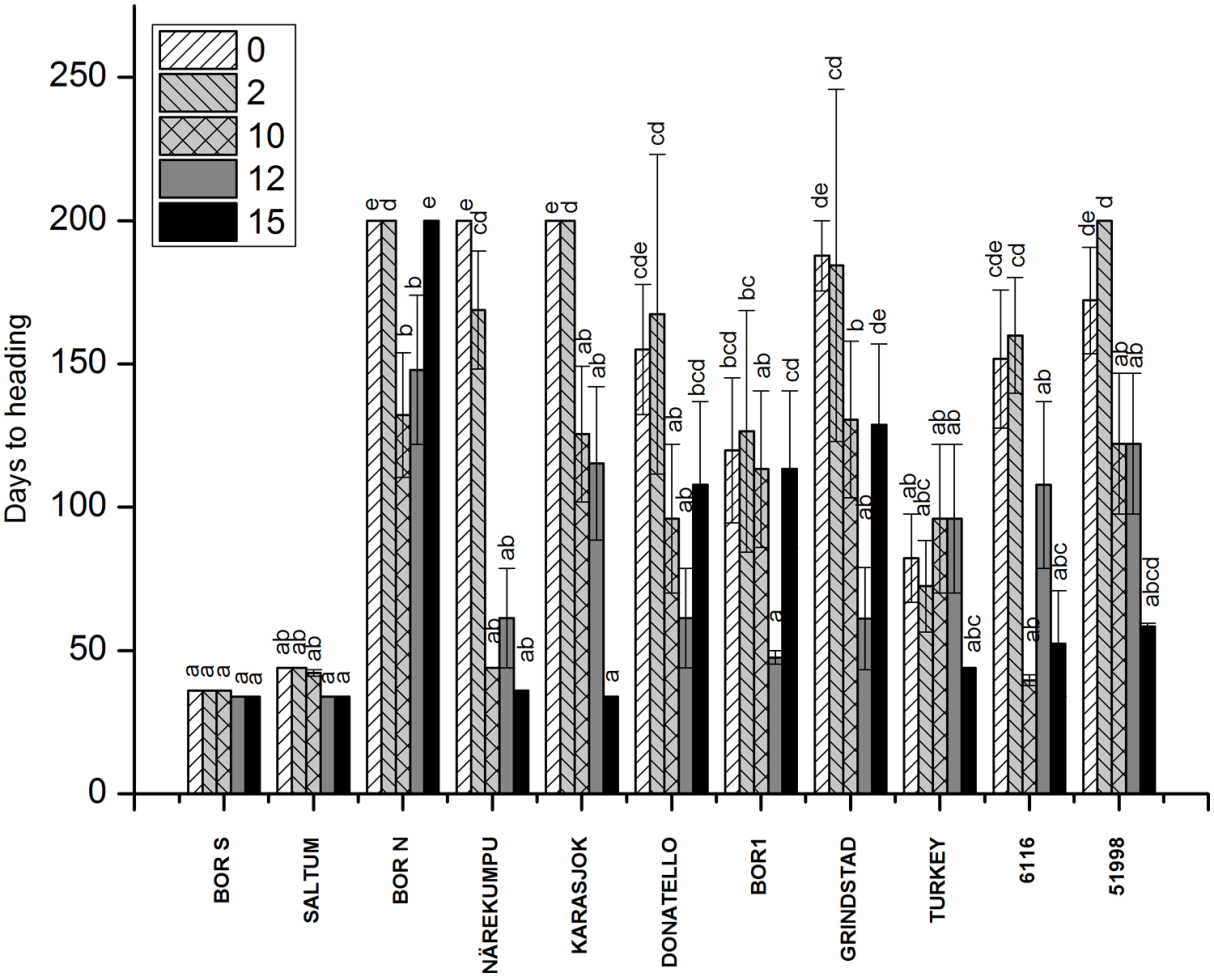
**The effect of vernalization on HD in timothy accessions (*n* = 9).** Plants were vernalized for 2, 10, 12, or 15 weeks in growth chamber [at 6/4°C (day/night), 8 h DL, control plants in 20/15°C (day/night), 12 h DL] and grown in 16-h DL in greenhouse for over 3 months. Some of the individual plants did not flower at all, and the HD was set for 200 for those plants. Data shows mean of three replicates of three plants ± SE. Distinct letters above bars indicate the significant differences in one vernalization treatment between accessions calculated by Tukey’s test.

**FIGURE 2:**
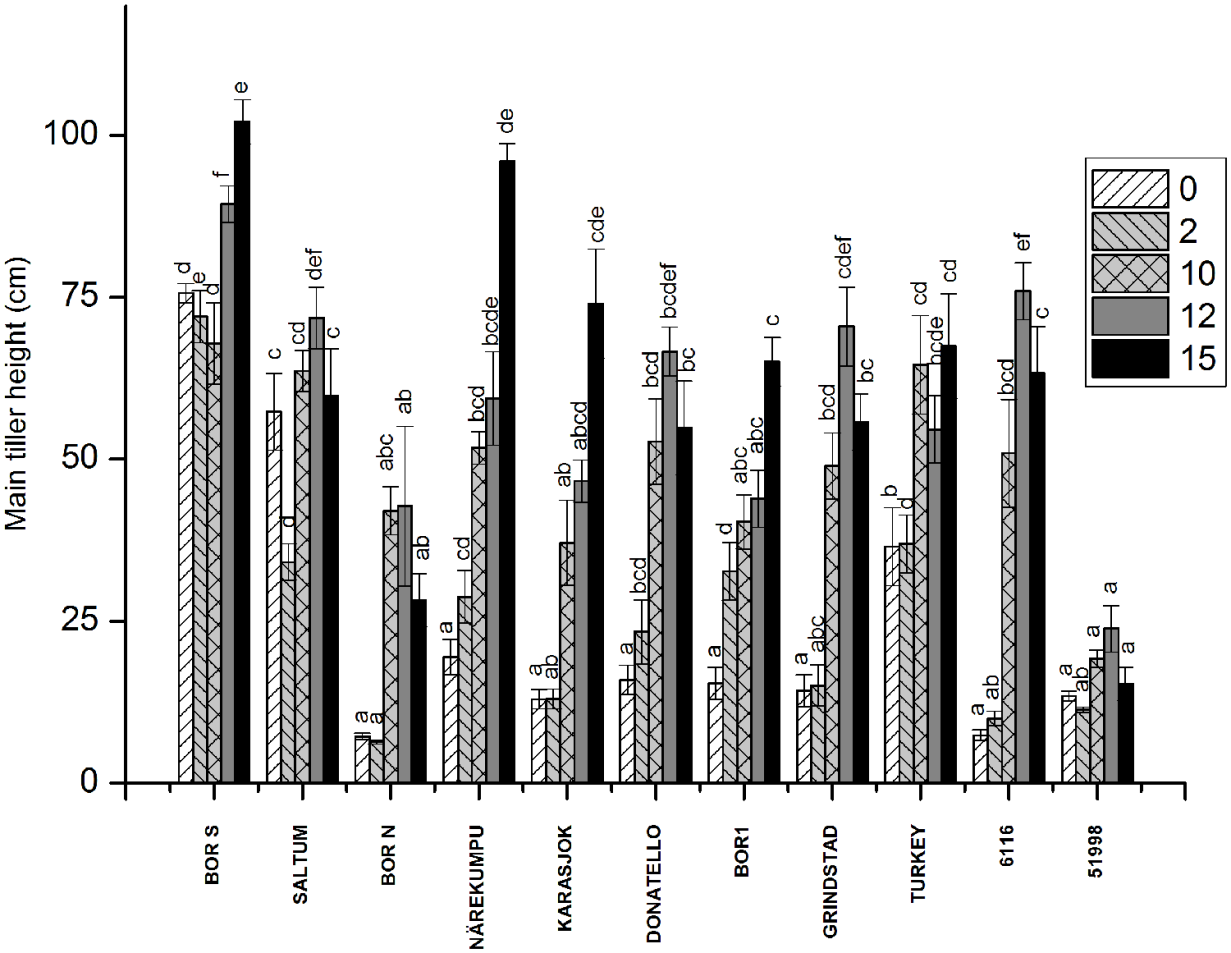
**The effect of vernalization on height of the main tiller in timothy accessions (*n* = 9).** Plants were vernalized for 2, 10, 12, or 15 weeks in growth chamber [at 6/4°C (day/night), 8 h DL, control plants in 20/15°C (day/night), 12 h DL] and grown in 16-h DL in greenhouse, and the height of the main tiller was measured after 3 weeks growth. Data shows mean of three replicates of three plants ± SE. Distinct letters above bars indicate the significant differences in one vernalization treatment between accessions calculated by Tukey’s test.

Wide variation in flowering time was found among the tested 11 accessions (*p* < 0.001; **Figure 1**). The most southern and northern accessions could be identified based on their divergent response to vernalization (**Figures 1** and **2**). Southern accessions (BOR S, Saltum) did not show vernalization response or requirement as the HD remained unchanged (HD ~40 days) irrespective of vernalization treatments. In comparison, Northern accessions (BOR N, Karasjok, Närekumpu) required 2–10 weeks vernalization for flowering. Moreover, HD occurred earlier in two northern accessions (Närekumpu, Karasjok) as the vernalization time got longer (**Figure 1**). The acceleration of heading was accession dependent being strongest in Närekumpu (from 200 to 40 days after 10 weeks) and slowest in BOR N (from 200 to 148 days in 12 weeks; **Figure 1**). After 15 weeks of vernalization the genotypic differences in HD were diminished (HD ca. 35 days) with exception of BOR N for which no flowering tillers were observed when plants were vernalized for 15 weeks and then grown in 16-h PP (**Figure 1**). The rest of the studied accessions were classified as intermediates as the HD decreased in response to vernalization (from ca. 75–40; **Figure 1**). These accessions, however, did not have an absolute vernalization requirement at this PP similarly to northern accessions and flowering tillers were eventually produced in non-vernalized plants within 60–90 days. The ANOVA showed significant differences between vernalization treatments, accessions and the interaction of these in HD (for all *p* < 0.001, **Table 2**).

**Table 2:** Summary analysis of variance (ANOVA) for HD in **(A)** Experiment 1, and **(B)** Experiment 2.

Source of variance	Degrees of freedom	Mean square	*p*-value
**(A)**			
Vernalization time	4	334.122	<0.001
Accession	10	277.661	<0.001
Vernalization time * accession	40	102.226	<0.001
**(B)**			
Vernalization time	2	90.443	0.001
PP	2	777.207	<0.001
Accession	3	58.891	0.004
Vernalization time * PP	4	78.892	<0.001
Vernalization time * accession	6	55.231	<0.001
PP* accession	6	55.029	<0.001
Vern. time* PP* accession	12	52.462	<0.001

The fulfillment of vernalization requirement was not clearly connected to FLN in timothy (FLN; **Supplementary Figure S2**). A relationship between the duration of vegetative stage of apices and FLN of the main tiller was not as clear, for instance BOR N remained vegetative and the FLN was also low (**Supplementary Figure S2**). Decreased leaf number was detected only in Närekumpu indicating that faster transition of apices in main tiller from vegetative to reproductive development resulted in fewer leaf primordia and thus less leaves (**Supplementary Figure S2**). In all other studied accessions, the number of developing leaves per tiller remained quite constant throughout vernalization treatments (**Supplementary Figure S2**). The ANOVA showed significant differences between the used vernalization treatments (*p* < 0.001), but the average of FLN in all studied accessions after 15 weeks vernalization was only 1, 5 leaves lower than in 0 weeks vernalized plants (**Supplementary Figure S2**).

### Interaction of Vernalization and Accession on Growth Characteristics

The release of stem growth was monitored by measuring the height of the individual plants 3 weeks after vernalization treatments (**Figure 2**). Rapid development and stem elongation was measured in the non-vernalized plants of southern accessions (BOR S, Saltum), in contrast to the majority of the northern accessions, which required vernalization for the initiation of stem elongation. Geographic origin did not, however, determine the response of height growth to vernalization although in two northern accessions (Närekumpu and Karasjok) the growth response to vernalization was notable (**Figure 2**). For example, accession 6116 originating from Italy required 10-weeks of vernalization for the initiation of rapid stem growth (**Figure 2**). On the contrary, in accession 51998 (England) stem elongation was arrested and was not affected by vernalization. Weakest response to height growth was seen in BOR N, which may be due to the restricting 16-h PP used in this experiment.

Timothy canopy consists of three tiller types and the proportions of these types vary depending on the physiological stage of the plant. Northern accessions were able to produce more tillers during vernalization, whereas southern accessions produced more tillers after vernalization (data not shown). At the end of the experiment each main tiller was categorized as VEG, ELONG, or GEN tillers. In BOR S all main tillers developed into GEN tillers without vernalization (**Table 3A**). Similarly, in Saltum and Turkey most of the main tillers in non-vernalized plants were generative (89%) whereas the northern accessions (BOR N, Närekumpu, Karasjok) were unable to produce GENs without vernalization in 16-h PP (**Table 3A**). In general, prolonged vernalization increased the proportion of GEN and ELONG main tillers so that after 15-weeks of vernalization none of the main tillers had remained VEG, expect in BOR N, where VEG tillers were present (**Table 3A**). The proportions of the main tiller type were significantly different between accessions and vernalization treatments (for both *p* < 0.001). The number of lateral tillers differed significantly between the studied accessions (*p* < 0.001, **Table 3B**). There were no interactions between vernalization time or observed vernalization response and total tiller number (*p* = 0.165). Most of the lateral tillers were VEG among accessions and vernalization treatments (**Table 3B**). In general, the number of GEN lateral tillers increased as the vernalization time prolonged (**Table 3B**). BOR S and Turkey produced significant proportion of GEN lateral tillers also in non-vernalized plants, whereas few flowering lateral tillers were observed in the rest of the southern and intermediate accessions (**Table 3B**). All three northern accessions (BOR N, Närekumpu, Karasjok) required 10 weeks vernalization for the development of GEN lateral tillers. The ANOVA showed significant differences between accessions in the number of VEG, ELONG, and GEN tillers (in all cases *p* < 0.001) and vernalization treatments (*p* = 0.005, *p* = 0.001, *p* < 0.001 respectively).

**Table 3:** **(A)** The percentage of the main tiller type (VEG, ELONG, and GEN) *n* = 9, and (**B**) the total number of side tillers and percentage of the side tiller type (VEG, ELONG, and GEN) in 11 timothy accessions (*n* = 9) after 0–15 weeks vernalization and 100 days (no change visible anymore) in greenhouse.

						Vernalization time (weeks)					

**(A)**

	0			2			10			12			15		
**Accession**	**VEG**	**ELONG**	**GEN**	**VEG**	**ELONG**	**GEN**	**VEG**	**ELONG**	**GEN**	**VEG**	**ELONG**	**GEN**	**VEG**	**ELONG**	**GEN**
BOR S	0	0	100	0	0	100	11	11	78	0	0	100	0	0	100
Saltum	0	11	89	0	11	88	0	0	100	0	0	100	0	11	88
BOR N	66	44	0	87,5	12,5	0	0	44	55	0	66	33	14	86	0
Närekumpu	11	89	0	33	44	22	0	0	100	0	11	88	0	0	100
Karasjok	11	89	0	66	33	0	11	33	55	0	44	55	0	11	88
Donatello	22	44	33	22	55	22	0	66	33	0	11	88	0	29	71
BOR1	11	33	55	55	44	0	44	44	11	0	0	100	0	37	62
Grindstad	66	33	11	44	44	11	0	55	44	0	0	100	0	50	50
Turkey	11	0	89	0	11	88	11	22	66	0	33	66	0	0	100
6116	37	50	13	16	33	50	0	0	100	12,5	25	62,5	0	0	100
51998	22	55	22	66	33	0	22	22	55	22	22	55	0	0	100

**(B)**

	0				2				10				12				15			
**Accession**	**Tillers**	**VEG**	**ELONG**	**GEN**	**Tillers**	**VEG**	**ELONG**	**GEN**	**Tillers**	**VEG**	**ELONG**	**GEN**	**Tillers**	**VEG**	**ELONG**	**GEN**	**Tillers**	**VEG**	**ELONG**	**GEN**

BOR S	80	56	20	24	133	71	15	14	42	31	33	36	51	35	20	45	82	61	7	32
Saltum	94	62	32	6	172	73	18	9	118	52	31	17	132	67	14	19	111	53	28	19
BOR N	142	80	20	0	189	95	5	0	68	97	3	0	108	49	44	6	151	46	53	1
Närekumpu	46	91	9	0	46	98	2	0	47	79	13	9	41	46	10	44	25	44	8	48
Karasjok	86	98	2	0	99	100	0	0	101	84	14	2	86	66	33	1	85	47	18	35
Donatello	49	76	20	4	61	67	33	0	114	58	30	12	81	58	17	25	120	46	42	13
BOR1	69	72	23	4	83	82	14	4	94	53	35	12	81	70	7	22	67	64	18	18
Grindstad	65	86	14	0	74	85	11	4	79	71	27	3	94	72	17	11	87	59	36	6
Turkey	74	46	27	27	88	67	18	15	67	25	28	46	94	52	22	26	100	22	41	37
6116	85	52	47	1	23	43	43	13	66	17	18	65	60	8	17	75	61	18	13	69
51998	100	92	7	1	103	98	2	0	121	55	32	12	104	51	32	17	154	51	36	13

### Interaction between Accession, Photoperiod and Vernalization on Flowering and Growth Characteristics

**FIGURE 3:**
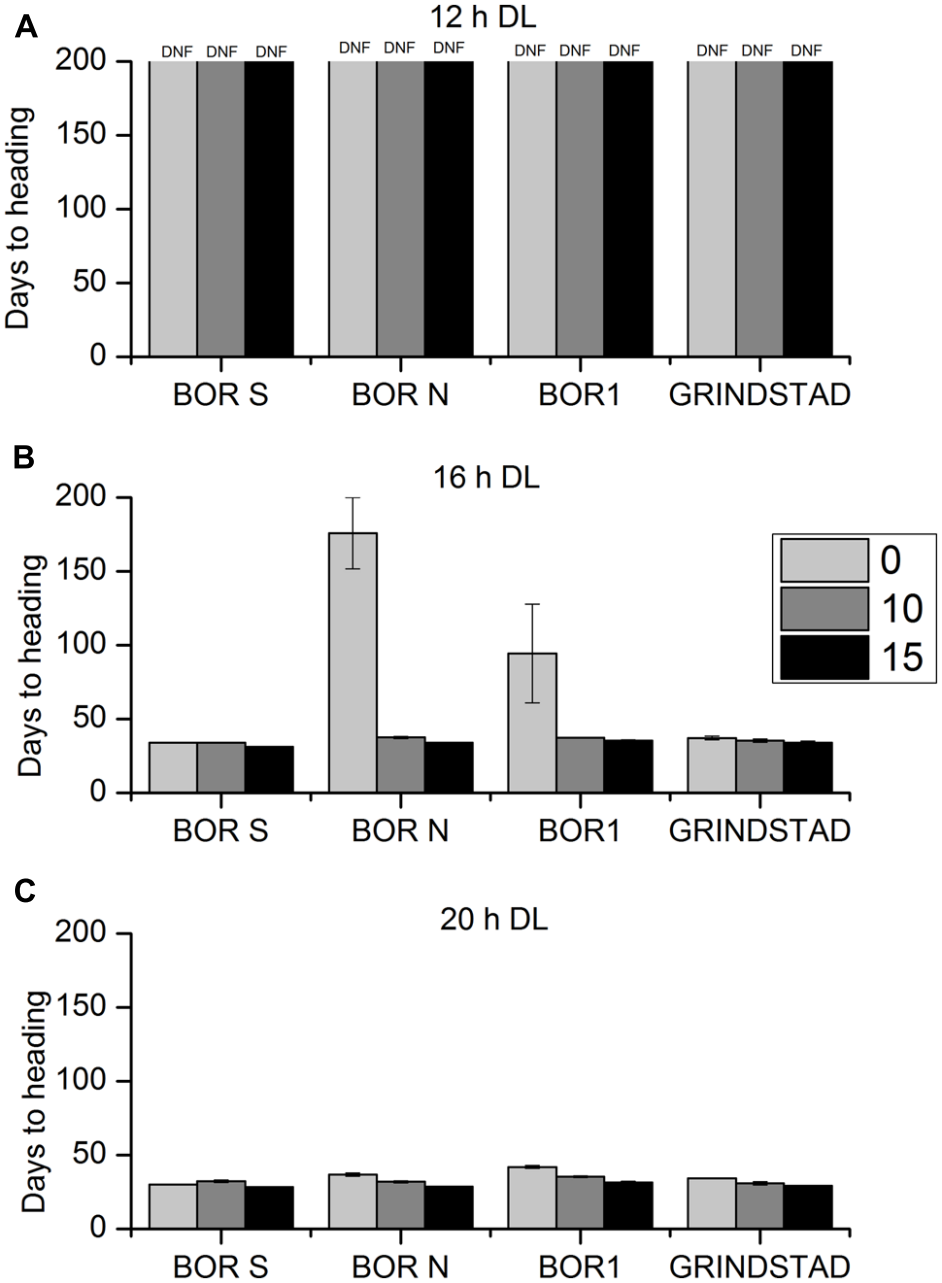
**The effect of different vernalization and PPs on heading time (HD) in four timothy accessions (*n* = 6).** Plants were vernalized 0 (light gray), 10 (dark gray), or 15 (black) weeks in growth chamber [at 6/4°C (day/night), 8 h DL; control plants in 20°C/15°C (day/night), 12 h DL] and transferred to **(A)** 12-h, **(B)** 16-h DL or **(C)** 20-h DL in greenhouse. Some of the individual plants did not head at all, and the HD was set 200 for those plants. Data shows mean of two replicates of three plants ± SE. DNF, did not flower.

The interaction between vernalization time and PP on flowering and growth characteristics was studied in four selected accessions having either strong, weak, or intermediate vernalization response (**Table 1**). At 12-h PP none of the tested plants produced flowering tillers, but flowering did occur in longer PPs (**Figure 3**). 20-h PP accelerated heading of all tested accessions by ca. 5 days compared to 16 h PP (**Figure 3**). In BOR N the HD shortened from 175 to 34 days at 20-h PP in non-vernalized plants (**Figure 3**) indicating that vernalization requirement was not obligatory at long enough DL. In fact, the genotypic differences in vernalization response were diminished at 20-h PP, but they existed at 16-h PP (**Figures 3B,C**). In BOR S and Grindstad neither vernalization nor change in PP from 16 to 20-h affected flowering time (**Figures 3B,C**). ANOVA showed significant differences between tested accessions, vernalization and PP treatments in HD (*p* = 0.004, *p* = 0.001, and *p* < 0.001, respectively, **Table 2**).

**FIGURE 4:**
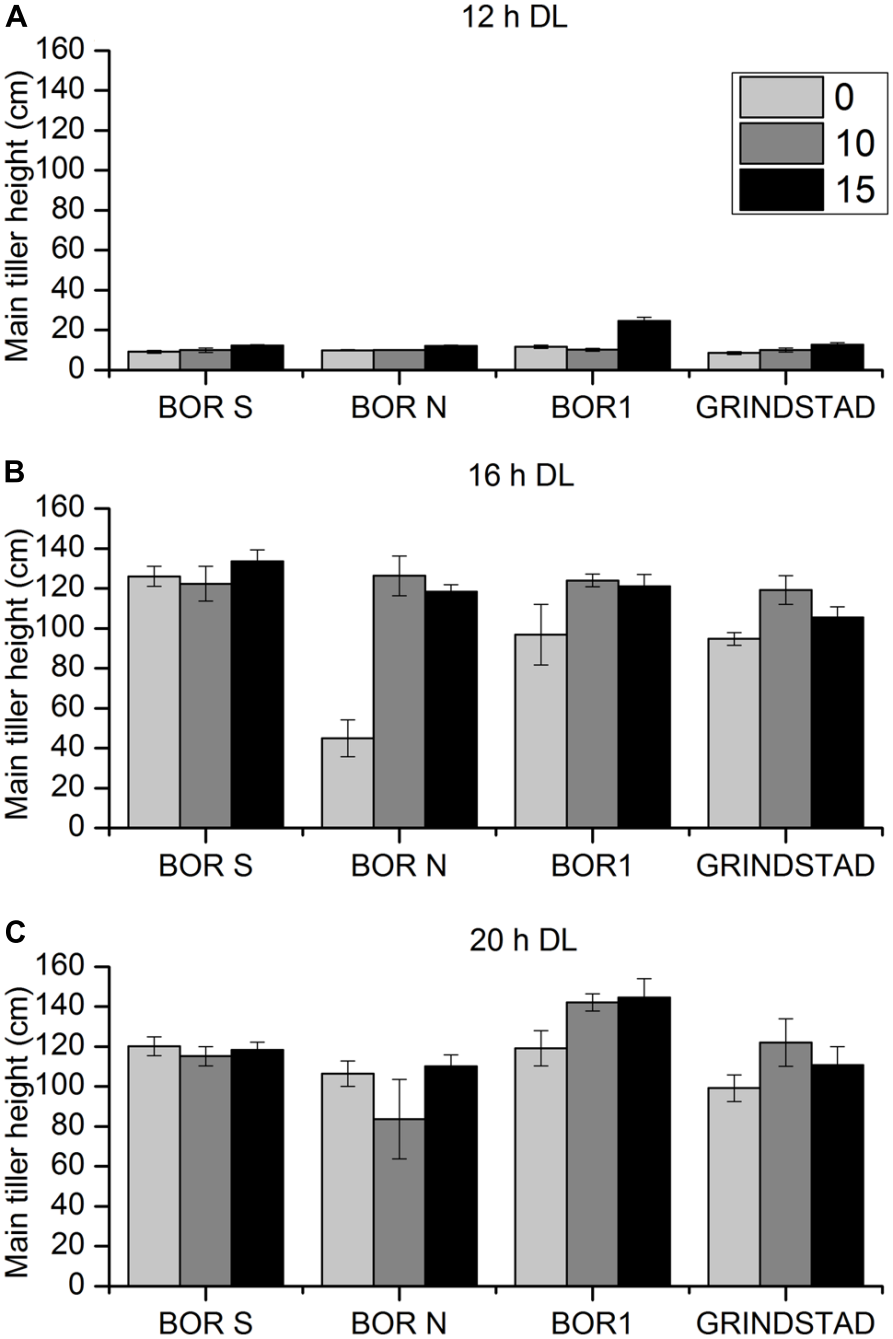
**The effect of different vernalization and PPs on main tiller height in four timothy accessions (*n* = 6).** Plants were vernalized 0 (light gray), 10 (dark gray), or 15 (black) weeks in growth chamber [at 6/4°C (day/night), 8 h DL; control plants in 20°C/15°C (day/night), 12 h DL] and transferred to **(A)** 12-h, **(B)** 16-h DL or **(C)** 20-h DL in greenhouse. Data shows mean of two replicates of three plants ± SE.

There were clear differences in height growth between accessions, vernalization and PP treatments (**Figure 4**, *p* = 0.006, *p* = 0.016, *p* = 0.001, respectively). Short day (12-h) arrested height growth in all studied accessions (**Figure 4A**). Modest growth at 12-h PP was, however, detected in BOR1 after long vernalization treatment (**Figure 4A**). In 16 h day length BOR N had strongest respond to PP, which was also seen in Experiment 1 (**Figures 2** and **4B**). Twenty hours PP was long enough to activate height growth also in non-vernalized BOR N, and the different vernalization treatments did not affect height in this PP (**Figure 4C**).

In 12 h PP only 15 weeks vernalized BOR1 was able to produce ELONG tillers (**Table 4A**). In 16-h PP only BOR N required vernalization for producing GEN tillers, whereas other accessions flowered without vernalization. However, BOR N was able to produce ELONG tillers without vernalization (**Table 4A**). In longer PPs development of tillers was shifted from VEG to GEN tillers in all accessions (**Table 4A**). The number of lateral tillers was highest in 12-h PP (**Table 4B**). All lateral tillers in 12-h PP were VEG, except BOR1 was able to produce few ELONG tillers after 15 weeks vernalization (**Table 4B**). The shift from VEG and ELONG tillers to GEN tillers was seen in most of the lateral tillers after all treatments in longer PP (**Table 4B**). Similar to Experiment 1, FLN did not correlate with the phenological development or vernalization response in timothy (**Supplementary Figure S3**).

**Table 4:** **(A)** The percentage of the main tiller type (VEG, ELONG, and GEN; *n* = 6), and **(B)** the total number of side tillers and percentage of the side tiller type in four timothy accessions (*n* = 6) after 0, 10, or 15 weeks vernalization and 100 days in greenhouse.

Vernalization time (weeks)															

**(A)**

		**0**			**10**			**15**		
**Accession**	**PP (h)**	**VEG**	**ELONG**	**GEN**	**VEG**	**ELONG**	**GEN**	**VEG**	**ELONG**	**GEN**
BOR S	12	100	0	0	100	0	0	100	0	0
BOR N	12	100	0	0	100	0	0	100	0	0
BOR1	12	100	0	0	100	0	0	0	100	0
Grindstad	12	100	0	0	100	0	0	100	0	0
BOR S	16	0	0	100	0	0	100	0	0	100
BOR N	16	0	100	0	0	0	100	0	0	100
BOR1	16	17	0	83	0	0	100	0	0	100
Grindstad	16	0	0	100	0	0	100	0	0	100
BOR S	20	0	0	100	0	0	100	0	0	100
BOR N	20	0	0	100	17	0	83	0	0	100
BOR1	20	0	0	100	0	0	100	0	0	100
Grindstad	20	0	0	100	0	0	100	0	0	100

**(B)**

		**0**				**10**				**15**			
**Accession**	**PP (h)**	**Tillers**	**VEG**	**ELONG**	**GEN**	**Tillers**	**VEG**	**ELONG**	**GEN**	**Tillers**	**VEG**	**ELONG**	**GEN**

BOR S	12	128	100	0	0	136	100	0	0	149	100	0	0
BOR N	12	122	100	0	0	132	100	0	0	121	100	0	0
BOR1	12	112	100	0	0	118	100	0	0	84	45	55	0
Grindstad	12	132	100	0	0	162	100	0	0	144	100	0	0
BOR S	16	66	14	0	86	75	33	25	41	77	36	9	55
BOR N	16	45	53	47	0	54	57	6	37	38	45	0	55
BOR1	16	68	18	24	59	66	24	0	76	69	17	12	71
Grindstad	16	78	18	15	67	47	0	49	51	72	0	39	61
BOR S	20	70	19	23	59	73	12	34	53	92	14	36	50
BOR N	20	53	19	6	75	63	38	27	35	70	26	19	56
BOR1	20	77	25	3	73	71	46	1	52	81	49	1	49
Grindstad	20	94	21	10	69	70	36	14	50	72	35	18	47

### Expression of Vernalization Genes after Vernalization and PP Treatments in Southern and Northern Accessions and Connection to Apex Development

**FIGURE 5:**
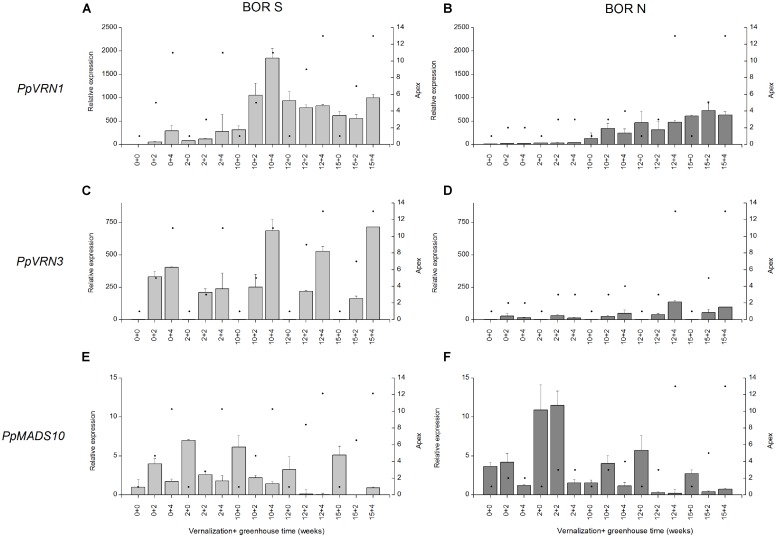
**(A,B)** *PpVRN1*, **(C,D)** *PpVRN3*, and **(E,F)** *PpMADS10* transcript levels in accessions BOR S and BOR N after 0–15 weeks vernalization [at 6/4°C (day/night), 8 h DL; control plants in 20/15°C (day/night), 12 h DL], and 0–4 weeks in a greenhouse (16 h DL). The expression levels are shown as the averages of three biological replicates ± SE. Relative expression is expressed as fold difference relative to transcript level of non-vernalized plants. Black spots on the right side *y*-axis describe the apex developmental stage in scale of Sweet et al. (1991).

The behavior of genes that regulate vernalization and PP flowering responses was studied in the most divergent northern (BOR N) and southern accessions (BOR S). The ability of southern accession to initiate stem elongation and produce flowering tillers without vernalization was associated with more rapid apex development and more intense transcript accumulation of *PpVRN1* and *PpVRN3* (**Figure 5**). After transfer from short day (12 h) to long day (16 h), apices of BOR S developed within two weeks from vegetative (apex 1) to generative stage (apex 6–10; **Figure 5**). This response occurred independently of vernalization treatment. The transition to generative growth in BOR S was accompanied by elevated transcript accumulation of *PpVRN1* (0 vern +4; **Figure 5A**) and *PpVRN3* (0 vern +2; **Figure 5C**). Reduced transcript levels for *PpMADS10* were detected after short vernalization (2 vern +2) and it occurred simultaneously with rapid development of apices (**Figure 5E**). In BOR S *PpVRN3* accumulation was detected in non-vernalized plants after transfer to 16-h PP and the level was slightly higher after 10–15 weeks vernalization (**Figure 5D**). *PpVRN1* transcript accumulation was responsive to vernalization (p < 0.001), with higher levels detected after 10–15 weeks vernalization versus after 0 or 2-weeks vernalization in both accessions (**Figures 5A,B**). In BOR N, development of apices was accelerated after 10-weeks of vernalization simultaneously with elevated transcript accumulation of *PpVRN1* (**Figure 5B**). However, *PpVRN1* and *PpVRN3* transcript accumulation was clearly less intensive in BOR N than in BOR S (**Figures 5A–D**), which was also shown by the ANOVA (*p* = 0.019 and *p* < 0.001, respectively). The accumulation of *PpMADS10* in BOR S was decreased after growth in greenhouse, but contradictory in BOR N this trend was not seen (**Figures 5E,F**).

**FIGURE 6:**
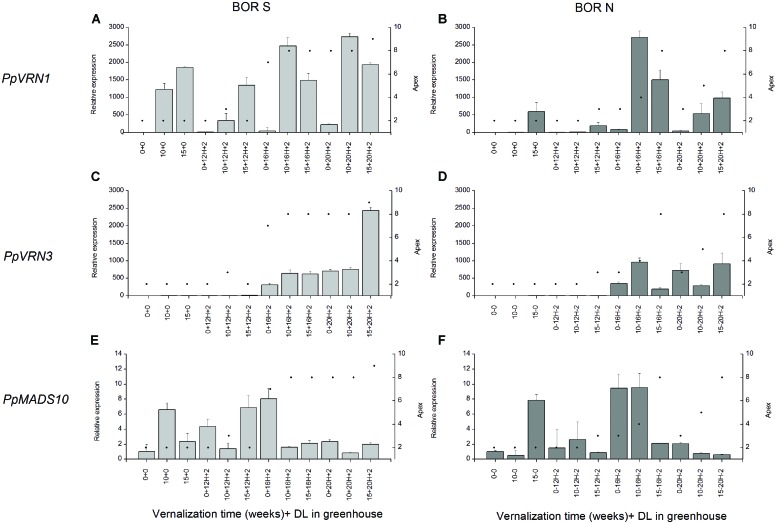
**(A,B)***PpVRN1*, **(C,D)** *PpVRN3*, **(E,F)** *PpMADS10* transcript levels in accessions BOR S and BOR N after 0, 10, or 15 weeks vernalization [at 6/4°C (day/night), 8 h DL; control plants in 20°C/15°C (day/night), 12 h DL], and 0 or 2 weeks in a greenhouse (in 12, 16, or 20-h DL). The expression levels are shown as the averages of three biological replicates ± SE. Relative expression is expressed as fold difference relative to transcript level of non-vernalized plants. Black spots on the right side *y*-axis describe the apex developmental stage in scale of Sweet et al. (1991).

We then examined gene expression after 2 weeks growth in different PPs following 0, 10, or 15 weeks vernalization (**Figure 6**). There was a correlation between elevated *PpVRN1* and *PpVRN3* expression levels and accession by treatment combinations that resulted in rapid flowering. However, there was higher *PpVRN1* accumulation in BOR S plants vernalized for 15 weeks and grown in 12-h PP without flowering. In BOR S *PpVRN1* transcript accumulated extensively during all vernalization treatments and the levels were lower when growing at 12-h (**Figure 6A**), whereas in BOR N decreased levels of *PpVRN1* transcript accumulated (**Figure 6B**). Increasing *PpVRN1* did not result in transcript accumulation of *PpVRN3* or development of GEN tillers at 12-h PP (**Table 4**, **Figures 6C,D**), whereas in 16-h and 20-h PP that occurred. Genotypic differences that were observed in apices development as well as in height growth (**Figure 4**) and tiller types (**Table 4**) at 16-h were associate with divergent transcript accumulation pattern of *PpMADS10* (*p* < 0.001; **Figures 6E,F**). In 16-h PP divergent accumulation of *PpMADS10* was seen in studied accessions; in BOR S the expression level was only high in non-vernalized plants, whereas in BOR N 15 weeks of vernalization was required for the low level (**Figures 6E,F**). At 20-h PP *PpMADS10* was at low level and genotypic differences were no longer visible (**Figures 6E,F**). At 20-h PP *PpMADS10* accumulation and the development of apices did not correlate as low levels of *PpMADS10* was detected although apices were at stage 8 in BOR S and 3 in BOR N (**Figures 6E,F**). Arrested apices development was associated with high levels of *PpMADS10* in both accessions (**Figures 6E,F**).

## Discussion

Climate change will dramatically affect forage production in the future. Adaptation to extended growing period and elevated temperatures in Northern hemisphere will require new cultivars, where the responses to PP and vernalization have been optimized for yield formation in novel growing conditions. Timothy ecotypes of different origin provide interesting genetic variation in their PP and vernalization responses that could be utilized in the future for breeding. In shorter PP the southern accessions with shorter critical PP and lack of vernalization response can produce higher dry matter (DM) yields as the tiller height growth, stem elongation or flowering are not restricted by environmental cues. In this study, vernalization was observed to progress stepwise so that first the main tillers were saturated and later the lateral tillers. Moreover, the development of GEN tillers in main and lateral tillers was more synchronized in southern accessions resulting in higher proportion of GEN tillers in canopy. On the contrary, in accessions 51998 (England) and 6116 (Italy) stem elongation was arrested and was not affected by vernalization. These plants remained short indicating probably adaptation for grazing. In silage production, the entire canopy is harvested several times during growing season and the proportion of tillers with true stem (either ELONG or GEN tillers) determines the quantity and quality of the yield. While in winter cereals the timing of flowering has a major role in adaptation (Shimizu et al., 2011), for forage grasses the major consideration with respect to cultivation is the influence of flowering behavior on growth rate and biomass accumulation in the short days of autumn.

In this study, wide variation in flowering time among timothy accessions in vernalization response was reported. Northern accessions typically showed a strong vernalization response, which was seen as decreased HD after vernalization, whereas southern accessions did not respond to vernalization and HD remained unchanged. In cereals rapid flowering without vernalization is typical for spring cultivars (e.g., Fowler et al., 1996; Danyluk et al., 2003) and it seems that in timothy both spring and winter type accessions exists within same species similar to *Arabidopsis* and cereals. In many species ecotypic variation in vernalization response has been seen, e.g., in *A. thaliana* (Grillo et al., 2013), *A. lyrata* (Quilot-Turion et al., 2013), barley (Loscos et al., 2014), *Brachypodium* (Ream et al., 2014), *Poa* sp. (Heide, 2001), and wheat (Iwaki et al., 2001). Fiil et al. (2011) reported that in timothy HD was correlated with geographic origin in non-vernalized plants, but after 15 weeks of vernalization differences were diminished. Also in this study flowering behavior was to some extent associated with the geographic origin of accessions studied. Vernalization response, expressed as a shortening of HD, was strongest in northern accessions and was absent in some southern accessions. It should be noted, however, that PP has the most important role in the regulation of flowering in timothy (Jokela et al., 2014) and that critical PP requirement varies between cultivars by their geographic origin (Heide, 1982, 1994). The 12 h PP has been shown to be too short for the flower initiation here, similar to earlier studies (e.g., Heide, 1982; Jokela et al., 2014). Here the most northern accession BOR N required longer vernalization for flowering in 16 h-PP. The genetic variation in HD diminished between accessions of different origin as the vernalization time or PP was long enough, and even BOR N was able to flower without vernalization in 20-h PP. Similar dependence on PP was not observed in *Arabidopsis* (Grillo et al., 2013) referring to a particular PP sensitivity of flowering in timothy, which has been observed to some degree also in *Brachypodium* (Ream et al., 2014). The possibility of SD induction for flowering during the pre-growth period in greenhouse can be rejected based on our earlier study where timothy plants were grown in 16-h PP following a similar pre- treatment, without vernalization, and were not able to flower (Seppänen et al., 2010). For the future research it should be noted that the experimental conditions, especially the greenhouse temperature and natural light, might have considerable effect on the growth of timothy plants. Here, the second experiment was conducted later in the spring when greenhouse temperature rose and PP was longer, which resulted in faster heading and taller plants compared to first experiment. However, the ability of accessions to flower was similar between them in both experiments. In addition, BOR S flowered constantly after ca. 45 days in both experiments, and thus it seems that the reaction to changing environmental conditions differs between accessions.

Final leaf number is useful characteristic to define the fulfillment of vernalization requirement of cereals and grasses (Frank and Bauer, 1995). This was first time to our knowledge when FLN was used to determine the vernalization fulfillment/response in forage grasses. However, it seems that FLN is unsuitable tool to define the vernalization saturation in timothy, because in several studied accessions the FLN was at constant level after all vernalization treatments despite the growth status. Only northern Närekumpu reacted similarly as winter cereals: when vernalization saturation was reached also the FLN was lower. It can be summarized that based on these experiments FLN cannot be used to determine timothy accessions having vernalization requirement. The typical growth habit for grasses is to produce more lateral tillers, and thus the relationship between FLN and vernalization might not be as significant as in winter cereals. So, whereas winter cereals show increasing leaf number with delayed flowering, FLN of timothy remains constant and there are instead increases in tiller number. In addition, the used experimental conditions may have kept some of the accessions in dormancy and thus, the leaf development had not started properly.

**FIGURE 7:**
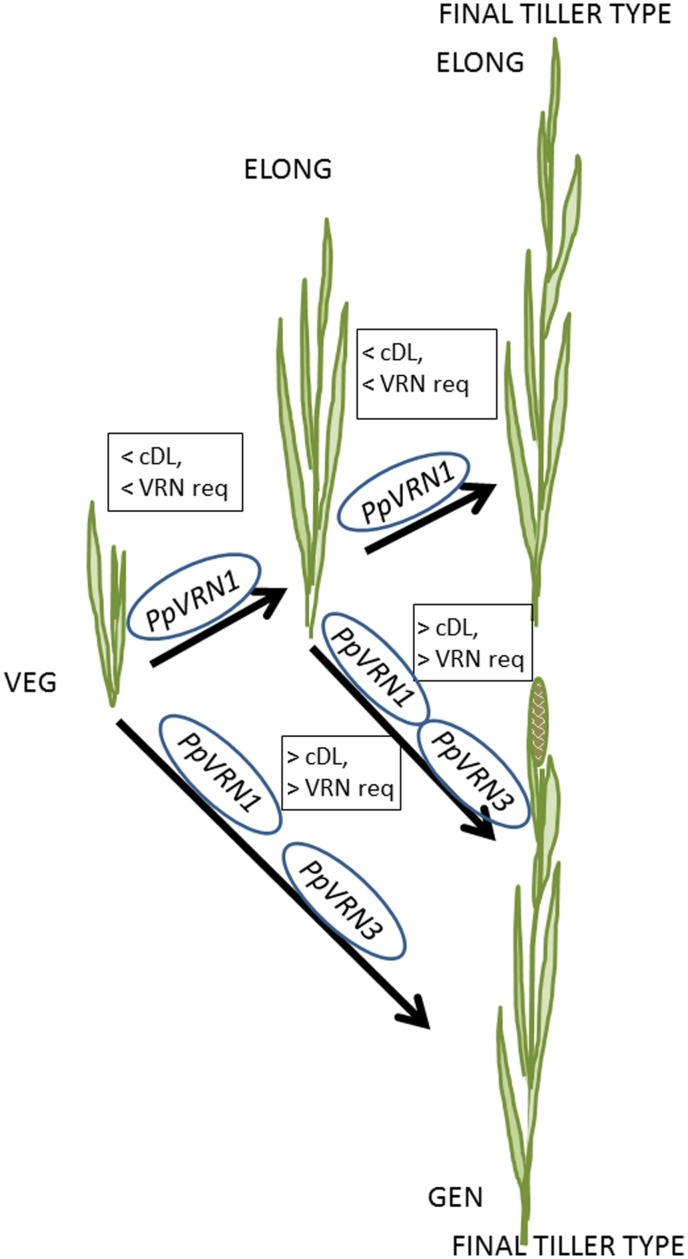
**A simplified schematic model to illustrate how critical day length (cDL) and vernalization requirement (VRN req) regulate the formation of vegetative (VEG), vegetative elongating (ELONG) and generative (GEN) tiller types in timothy.** The final tiller type is either ELONG or GEN tiller. In addition, the proposed roles of *PpVRN1* and *PpVRN3* in tiller formation are presented.

A clear connection between flowering and expression of *PpVRN1* and *PpVRN3* was detected in this study in timothy (**Figure 7**). In temperate cereals *VRN1* is activated by vernalization, which in turn blocks *VRN2* (Trevaskis et al., 2006; Hemming et al., 2008), and *VRN3* acts as an integrator of vernalization and PP pathways (reviewed, e.g., Distelfeld et al., 2009; Kamran et al., 2014). Ream et al. (2014) suggested that there exists a positive feedback loop between *VRN1* and *VRN3/FT*, and it is conserved throughout *Pooideae* species including *Brachypodium*. Recent study by Deng et al. (2015) revealed that PP and vernalization response pathways have a direct connection, and moreover *FT1* (*VRN3*) is a direct target of *VRN1*. Ergon et al. (2013) proposed that, however, the role of *VRN1* in outcrossing forage grass species may be less important than in cereals. This hypothesis was supported by the present results and our earlier study, where we suggested that *PpVRN1* alone is insufficient to trigger flowering stimulus, and *PpVRN3* is required for flowering (Jokela et al., 2014). Diallo et al. (2012) suggested that *TaFT1*, homolog of *PpVRN3*, is involved at the last stages of the transition to reproductive growth in wheat, and it is not required after the flowering initiation. The major role of *VRN3/FT* genes connected to PP sensitivity has been shown earlier also, e.g., in barley (Hemming et al., 2008), oats (Nava et al., 2012), and wheat (Diallo et al., 2012), where the expression of *TaFT1* was significantly higher in spring cultivar. In our earlier study, we speculated that the PP sensitivity in two studied cultivars could be seen in the expression levels of *PpVRN3* (Jokela et al., 2014). Similar results were obtained in the present study, where the *PpVRN3* expression levels were constantly higher in BOR S compared to BOR N, probably due the potential role of *PpVRN3* as an integrator of vernalization and PP pathways. Interestingly, here the expression peak of *PpVRN3* was detected in BOR S plants grown 2 weeks in 16 and 20 h PP, also without vernalization.

Studies done with cereals have shown that especially rapid flowering accessions holding dominant alleles of *VRN1* and *FT* (*VRN3*) can bypass the vernalization requirement (Yan et al., 2006; Faure et al., 2007). Based on the results in this paper, it seems that this kind of bypassing the vernalization requirement is also possible in timothy if PP is long enough. Taken together, these observations enable the speculations of the crucial role of PP in the flowering of the timothy both at physiological and molecular level. The transcript accumulation of *PpVRN1* and *PpVRN3* were at different levels, and the vernalization saturation occurred at divergent rate in two separate experiments. Although, the vernalization saturation was always connected to higher expression of the studied genes, and if plants were not saturated, also the levels of *PpVRN1* and *PpVRN3* remained low. The allele form of *VRN1* has been shown to classify cereal types to spring (dominant), and winter (recessive) types (Fu et al., 2005; Chu et al., 2011). Further, vernalization has promoting effect on the level of active state of *TaFT1* chromatin in winter types, whereas in spring types vernalization reduces it (Diallo et al., 2012). In timothy accessions, on the contrary, vernalization does not seem to have similar effect on the expression of *PpVRN3*. Hence, the more detailed analysis and grouping timothy accessions to spring and winter types would require allelic and epigenetic studies of *PpVRN1* and *PpVRN3*.

In *Arabidopsis* genetic variation and linkage of flowering time and adaptation has been analyzed in locally adapted populations (Grillo et al., 2013). In general, *Arabidopsis* ecotypes can be characterized either as winter annuals, summer annuals germinating in spring and flowering without vernalization, or overwintering as rosettes requiring vernalization (Alonso-Blanco and Koornneef, 2000). Grillo et al. (2013) found that winter annuals originating from Sweden failed to flower without vernalization, whereas populations from Italy flowered. Moreover, the northern populations had longer time to flower after vernalization, i.e., they could be characterized as late flowering types (Grillo et al., 2013). The northern *Arabidopsis* accessions have strict vernalization requirement for flowering, whereas in timothy PP can diminish vernalization requirement, as it has been shown in this study. Mutant studies have revealed that in *Arabidopsis* winter annuals allelic variation in *FRI* and *FLC* is the main regulators of flowering (Lee et al., 1993; Clarke and Dean, 1994; Johanson et al., 2000; Lempe et al., 2005). In the presence of functional *FRI* alleles, latitudinal decline in flowering time is correlated with *FLC* expression (Caicedo et al., 2004; Lempe et al., 2005; Shindo et al., 2005). Results on the QTL mapping of F2 populations produced by crossing northern and southern *Arabidopsis* populations show that the genetic variation in flowering time was co-localized to *FLC* locus (Grillo et al., 2013). In timothy divergent transcript accumulation of *PpMADS10* was seen between accessions especially at conditions where the response to vernalization was different. The variation in HD in timothy was diminished in long enough PP or vernalization treatments. Furthermore, in growing conditions, where ecotypical differences in flowering behavior between BOR S and BOR N were not seen differences in *PpMADS10* were also absent. It is possible that the variation in *PpMADS10* expression in southern and northern timothy accessions seems to be comparable to the variation of *FLC* expression in *Arabidopsis* ecotypes of different origin. It should be noted, however, that there was also a strong interaction between developmental stage of the shoot apex and *PpMADS10* expression level. In most cases at 16- and 20-h PP low *PpMADS10* levels were detected in the apices at the reproductive stage, so low *PpMADS10* expression might be a consequence of reproductive development rather than the cause.

Our results provide more information on the regulation of flowering and canopy structure in perennial forage grass, timothy. It seems that PP is the key regulator in the initiation of flowering. In addition, in northern accessions also vernalization has important role. However, after long enough vernalization or PP treatments differences in HD between accessions diminished. The expression of flowering promoting genes, *PpVRN1* and *PpVRN3*, were connected to vernalization saturation, and flowering. Both physiological and molecular observations indicate that spring and winter types can be found also in timothy.

## Author Contributions

VJ and MS planned the study. VJ carried out the experiments and analyzed the data. All authors wrote, read and approved the final manuscript.

## Conflict of Interest Statement

The authors declare that the research was conducted in the absence of any commercial or financial relationships that could be construed as a potential conflict of interest.

## Supplementary Material

Figure S1 **The tiller types of timothy, (A) generative (GEN), **(B)** vegetative elongating (ELONG), **(C)** vegetative (VEG) tiller**. Photograph: Perttu Virkajärvi.

Figure S2 **The effect of vernalization on final leaf number (FLN) in timothy accessions (*n* = 9)**. Plants were vernalized for 2, 10, 12, or 15 weeks in growth chamber [at 6/4°C (day/night), 8 h DL; control plants in 20/15°C (day/night), 12 h DL] and grown in 16-h DL in greenhouse for over 3 months. FLN was calculated at the end of the experiment. Data shows mean of three replicates of three plants ± SE. Distinct letters above bars indicate the significant differences in one vernalization treatment between accessions calculated by Tukey’s test.

Figure S3 **The effect of different vernalization and PPs on FLN in four timothy accessions (*n* = 6)**. Plants were vernalized 0 (light gray), 10 (dark gray), or 15 (black) weeks in growth chamber [at 6/4°C (day/night), 8 h DL; control plants in 20 /15°C (day/night), 12 h DL] and transferred to **(A)** 12-h, **(B)** 16-h DL or **(C)** 20-h DL in greenhouse. FLN was calculated at the end of the experiment. Data shows mean of two replicates of three plants ± SE.

## Abbreviations

ELONG: vegetative elongating tiller
FLN: final leaf number
GEN: generative tiller
HD: heading date
LAI: leaf area index
PP: photoperiod
VEG: vegetative tiller
VRN: vernalization genes.
