# Supplementary material for: Genetic variation in the flowering and yield formation of timothy (Phleum pratense L.) accessions after different photoperiod and vernalization treatments
Source: Front Plant Sci. 2015 Jun 30;6:465. doi: 10.3389/fpls.2015.00465 (PMC4485155; doi:10.3389/fpls.2015.00465)
Supplement: Supplementary file 1 [file Table_1.PDF]

Supplementary material. Species specific primers used in q-RT-PCR analysis.

Gene ID	Primer sequence
Actin_F	ACTGGGACGACATGGAGAAG
Actin_R	CTGTTAGCCTTGGGGTTCAG
PpVRN1_F	GAGCGGTATGAGCGCTACTC
PpVRN1_R	TTCAGATTCGGTTGAAATGAGA
PpMADS10_F	AACTACAGCGAAAGGGCTCA
PpMADS10_R	AGCTTGCCAGCTGTTGGTAT
PpVRN3_F	GGACAACTGGTGCTTCCTTC
PpVRN3_R	GAGGGCTCTCGTAGCACATC
