# Supplementary figures and images for: Genetic variation in the flowering and yield formation of timothy (Phleum pratense L.) accessions after different photoperiod and vernalization treatments
Source: Front Plant Sci. 2015 Jun 30;6:465. doi: 10.3389/fpls.2015.00465 (PMC4485155; doi:10.3389/fpls.2015.00465)

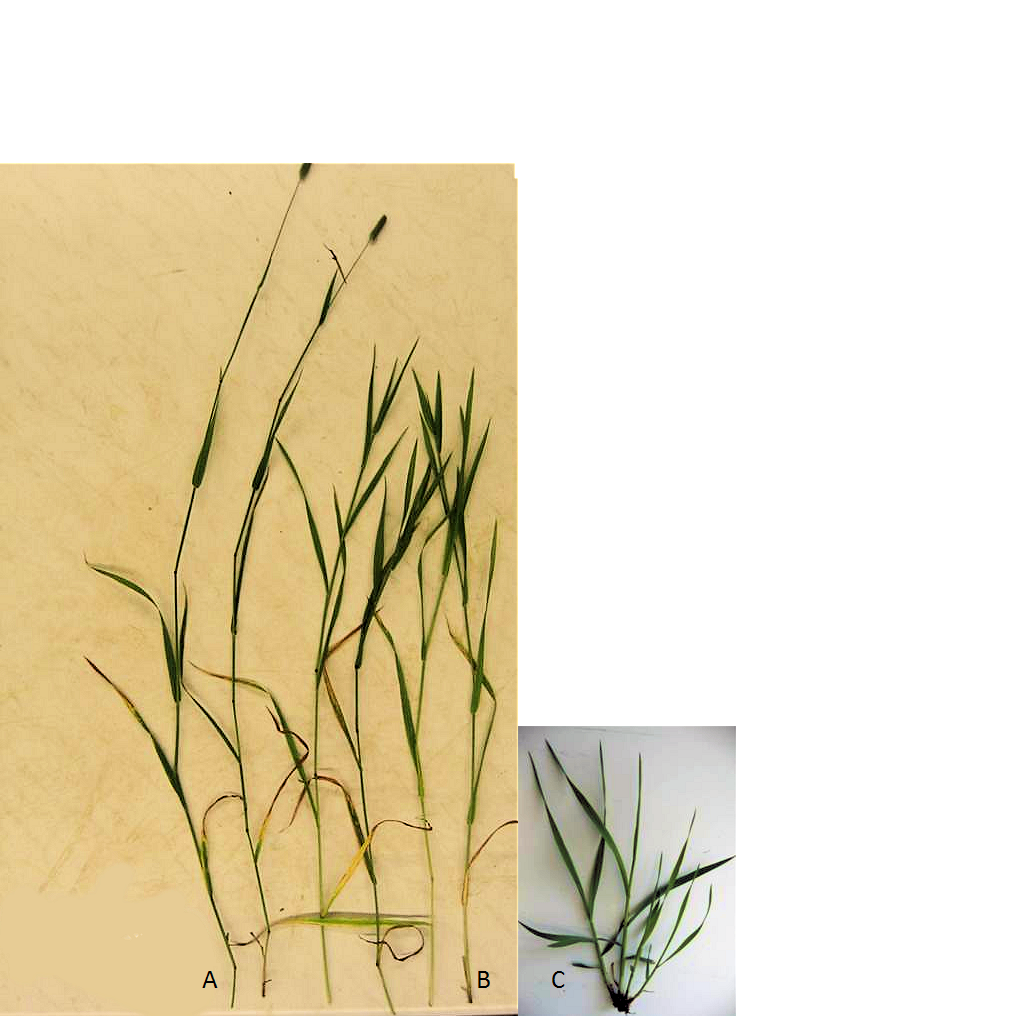

Supplement: Figure S1 — The tiller types of timothy, (A) generative (GEN), (B) vegetative elongating (ELONG), (C) vegetative (VEG) tiller. Photograph: Perttu Virkajärvi. [file Image_1.TIF]
